# Heavy metals and potential health risk assessment of *Lactuca sativa* and *Daucus carrota* from soil treated with organic manures and chemical fertilizer

**DOI:** 10.1007/s10661-024-12687-y

**Published:** 2024-05-10

**Authors:** O. E. Aina, L. L. Mugivhisa, J. O. Olowoyo, C. L. Obi

**Affiliations:** 1 Department of Biology and Environmental Science: School of Science and Technology, Sefako Makgatho Health Sciences University, Pretoria, South Africa; 2 Department of Health Sciences and The Water School, Florida Gulf Coast University, Fort Myers, FL 33965 USA

**Keywords:** Accumulator, Excluder, Carcinogenic, Non-carcinogenic, Soil management

## Abstract

The large-scale production of food crops with heavy application of chemical fertilizers in the effort to meet the astronomical increase in food demands may be counterproductive to the goal of food security. This study investigated the effect of different soil treatments on the levels of heavy metals (Cr, Cu, Fe, Ni, Pb, and Zn) in two types of vegetables *Lactuca sativa* (lettuce) and *Daucus carrota* (carrot). The potential carcinogenic and non-carcinogenic health risks from their consumption were also evaluated. Planting experiment was set up in a randomized block design, with different soil treatments of soil + cow dung (CD), soil + sewage sludge (SS), soil + chemical fertilizer (nitrogen-phosphorus-potassium (NPK)), and untreated soil (UNTRD). The vegetables were harvested at maturity, washed with distilled water, and subjected to an acid digestion process before the levels of heavy metals were measured by inductively coupled plasma spectrometry (ICP-MS). The mean concentrations of the metals in the vegetables across all treatments were below the maximum permissible limits. The pattern of heavy metal accumulation by the vegetables suggested that the lettuce from SS treatment accumulated higher concentrations of heavy metals like Cr (0.20 mg/kg), Cu (3.91 mg/kg), Ni (0.33 mg/kg), and Zn (20.44 mg/kg) than carrot, with highest concentrations of Fe (90.89 mg/kg) and Pb (0.16 mg/kg) recorded in lettuce from NPK treatment. The bioaccumulation factor (BAF) showed that lettuce, a leafy vegetable, has bioaccumulated more heavy metals than carrot, a root vegetable. The BAF was generally below the threshold value of 1 in both vegetables, except in lettuce from NPK and CD treatments and carrot from NPK treatments, with BAF values of 1.6, 1.69, and 1.39, respectively. The cancer risk assessment factors were well below the unacceptable maximum range of 10^−4^ suggesting that consuming these vegetables might not expose an individual to potential risk of cancer development. The hazard quotient estimations were below the threshold values of 1 for all heavy metals; however, the hazard index (HI) values of 1.27 and 1.58 for lettuce from NPK and SS treatments indicate a potential non-carcinogenic health risk to consumers from intake of all the heavy metals.

## Introduction

Food and Agriculture Organization (FAO) defined food security as a state where all people have physical, social, and economic access to sufficient, safe, and nutritious food which meets their dietary needs and food preferences for an active and healthy life (FAO et al., 2021). However, challenges to food security include urbanization and industrialization resulting in the reduction of agricultural land for planting purposes, leading to overcultivation of the available land area. This has resulted in the loss of soil fertility, reduced quantity, and low quality of food crops. Nevertheless, the demand for food has continued to skyrocket due to astronomical increase in human population. Consequently, farmers and other growers now employ different soil management techniques including application of chemical fertilizers and organic manures in the production of food crops.

Heavy metals are a major environmental pollutant of interest due to their association with a wide range of health problems. Their occurrence in the environment is due to natural causes and anthropogenic activities. Agricultural practices, especially soil management techniques for crop productions, have been reported as some of the major anthropogenic activities that contribute to the environmental accumulation of heavy metals (Goss et al., 2013; Kumar Bhatt et al., 2019; Vácha, 2021).

Large-scale crop production which relies heavily on intensive application of chemical fertilizers is a major source of environmental pollution such as soil and groundwater acidification, salination, and alga bloom pollution of other water bodies (Chauhan & Mathur, 2020; Cui & Shoemaker, 2018; Singh et al., 2020). Also, repeated and consistent application of chemical fertilizers over time contributes to accumulation of heavy metals in crop soil (Jiao et al., 2012; Khan et al., 2018). This has led to a substantial paradigm shift to the use of organic manures because of their ability to improve soil physical, chemical, and biological properties such as the pH, electrical conductivity, organic matter, humus, and aeration which in turn aids nutrient solubility and availability to plant (Kumar Bhatt et al., 2019). It is also believed that food crops from soil treated with organic manures are more nutritious, safer, and less contaminated (Liu et al., 2013). Furthermore, organic manure like sewage sludge is reportedly rich in macronutrients that play crucial roles in the development, growth, and nutrient setting of crops (Hamdi et al., 2019). Likewise, cow dung is reported to improve soil properties such as organic matter, microbiome, and water retention capacity, thereby reducing leaching and nutrient runoff which are subsequently made available to crops (Liu et al., 2017; Siedt et al., 2021).

However, the application of organic manure can also be a major source of pollutants especially heavy metals and their environmental accumulation. For instance, sewage sludge may contain heavy metals coming from sources like industrial waste and runoff from roofs, roads, and parking lots (Goss et al., 2013). Similarly, dungs from animals that have ingested heavy metal-contaminated feed or feeds containing spiked amount of trace elements for lameness treatment may be a source of heavy metal contamination in agricultural soil (Aylaj et al., 2019; Goss et al., 2013).

Although some of these heavy metals are important cofactors in biological, physiological, and metabolic processes in humans, most of them however have no benefit, and exposure to high concentrations can lead to serious health hazard (Zhou et al., 2016). Some of these heavy metals have been classified as human carcinogens by the International Agency for Research on Cancer (Lyon, 2010; Pipoyan et al., 2020). Apart from carcinogenic diseases, the list of health hazards associated with heavy metals includes non-carcinogenic diseases such as cardiovascular, degenerative, neurological, hematological, and reproductive diseases (Esposito et al., 2019; He et al., 2022). Also, exposure to high doses of heavy metals over a long period has been associated with kidney and lung damage (Taiwo et al., 2022). The ability of heavy metals to bioaccumulate in plants has become a great concern because of their major health implication on humans who may become exposed to high concentrations through food chain (Adani et al., 2022; Olowoyo et al., 2012).

Vegetables are reportedly great sources of nutrients and many other health benefits, which make them an important part of human daily diet (Esposito et al., 2019; Guadie et al., 2021). Fresh produce like *Lactuca sativa* and *Daucus carrota* subsequently referred to as lettuce and carrot are very popular and commonly consumed vegetables across the world due to their many nutritional and health benefits (Ansorena et al., 2012; Cruz et al., 2014). These vegetables have been reported for their abundant nutritional contents of secondary metabolites such as lycopene, flavonoids, antioxidants, vitamins, and other major macronutrients (Gupta et al., 2005; Uusiku et al., 2010).

South Africa is classified as self-sufficient in the production of lettuce and carrot, that is, they produce adequate quantities for consumption with the surplus exported to other Southern African countries. The latest 10-year data profile report on the vegetable market value chain from the Department of Agriculture, Land Reform and Rural Development, shows that South Africa consumes an average of 30,636 and 174,717 tons of lettuce and carrot, respectively (DALRRD, 2021a, 2021b). The data also show that although South Africa is not a major exporter of these two vegetables, they nonetheless represent about 0.1% and 0.5% of the world exports of lettuce and carrot, respectively. Vegetable production contributes substantially to the global economy as well as alleviating poverty and unemployment in developing countries. Several factors ranging from short cycle production period to diverse health promoting nutritional compounds are responsible for the massive production of vegetables (Głąbska et al., 2020). Some of the health benefits of vegetables include reduced risk of developing cancers and cardiovascular diseases (Menni et al., 2021). The recommended daily intake of vegetable is put at 240 g day^−1^ (Herforth et al., 2019).

Different soil management techniques such as the application of organic and inorganic fertilizers as soil conditioners are used in the cultivation of vegetables (Hammad et al., 2020; Shah & Wu, 2019). These soil conditioner materials are reported to contain different pollutants including heavy metals that can bioaccumulate in plant tissues (Hammad et al., 2020; Nawab et al., 2019). The application of organic manures and chemical fertilizers has been reported as one of the sources of heavy metals in food crops (NING et al., 2017). Vegetables particularly have been reported for their remarkable ability to bioaccumulate heavy metals from the soil in their tissues (Alimohammadi et al., 2020; UNVER et al., 2015). The health hazard associated with the unintentional and unconscious overdose ingestion of these metals from food crops has become a major concern to scientists, and this has encouraged a lot of agronomy research studies.

Evidence from literatures suggests that there is no conclusive assertion on which soil treatments increase or reduce the chances of heavy metal contamination of food crops. This is because the uptake of trace elements from the soil by plant is influenced by many factors such as soil pH, organic matter, electrical conductivity, and plant species, in addition to the concentration of elements in the soil (Aina et al., 2019; Khan et al., 2014). Therefore, the current study assessed the effect of different soil treatments and types of vegetables on the uptake and bioaccumulation of heavy metals by vegetables. We hypothesized that vegetables cultivated on organic manures bioaccumulate higher levels of heavy metals compared to the ones grown on chemical fertilizer treated soil. The objectives are to compare the heavy metal concentrations in different types of vegetables, and from different soil treatments, as well as evaluate potential heavy metals associated health hazard that may emanate from their consumption.

## Methodology

This study was conducted at Sefako Makgatho Health Sciences University located in the northern part of Pretoria, one of the cities in Tshwane Metropolitan Municipality in South Africa (250 37′ 8″ S and 280 1′ 22″ E). Pretoria is the administrative capital of South Africa. It has a monsoon influenced humid subtropical climate with long hot rainy summers and short, dry, and mild winters. Pretoria experiences an average annual temperature of 18.7 °C (65.7 °F) and 675 mm (26.6 in) average annual rain (Beraki et al., 2013). The planting experiments were conducted at the production unit of the university, in a net fenced enclosure to prevent intrusions from animals and unauthorized persons. This area is used for a lot of experimental planting and some other research activities in the university.

### Experimental design and planting

This study was conducted on lettuce and carrot using two organic manures (sewage sludge and cow dung animal manure) and mineral fertilizer (NPK). The sewage sludge was collected from Daspoort Sewage Plant, Marabastad, a governmental organization located in the central city of Tshwane, South Africa. The cow dung was collected from the livestock farm at De-Wilt, Brits Road Ga-Rankuwa, located in the north of the city of Tshwane, South Africa. The nitrogen-phosphorus-potassium fertilizer with a weight distribution of N_2_P_3_K_2_, i.e., 40% nitrogen, 60% phosphorus, and 40% potassium, manufactured by Omina Fertilizers Johannesburg, South Africa, was purchased from a registered nursery marketer (Plantland Nursery), Longmore and Old Brits Road, Akasia—Pretoria South Africa. The planting was done using a total of 96 pots divided into two equal numbers for each vegetable and further subdivided into 12 pots per treatment (sewage sludge treated soil (SS), cow dung treated soil (CD), NPK treated soil (NPK), and untreated soil (UNTRD)). The soil quantity and manure dry weight used in this study were adapted from previous studies by Aina et al. (2018) and Li et al., (2021a, 2021b). Every pot from the treatment groups was filled with a thoroughly mixed 5 kg of sand loamy soil and 0.15 kg of amendment, while pots from the untreated group were filled with ordinary 5.15 kg of sand loamy soil. Also, the 0.15 kg weight of amendments used was compared to the recommended dose of NPK fertilizer for vegetable cultivation (Hammad et al., 2020). The seedlings of lettuce and carrot which were raised at self-propagated nursey were introduced into the pots 2 weeks after germination. The plants were exposed to the same normal environmental condition and irrigated with the same quantity of portable water enough to moisten the soil to prevent nutrient runoff and NO_3_^−^ leaching, and reduce experimental bias of soil properties (Table 1) (Daliakopoulos et al., 2016; Thompson et al., 2020).

**Table 1:** The physicochemical properties of soil

Treatments	Properties					
	Sand (%)	Silt (%)	Clay (%)	OM (%)	EC (µscm^−1^)	pH (H_2_O)
UNTRD	68.9	17.3	13.8	1.42	8.04	7.39 ± 0.06
NPK	ND	ND	ND	1.36	14.72	7.18 ± 0.08
CD	ND	ND	ND	2.13	9.14	7.41 ± 0.04
SS	ND	ND	ND	2.42	22.43	7.12 ± 0.07

*UNTRD* untreated soil, *NPK* NPK soil, *CD* cow dung soil, *SS* sewage sludge oil, *ND* not determined

### Sample preparation and digestion process

The vegetables were harvested at maturity of 60 and 110 days for lettuce and carrot, respectively, rinsed with distilled water, with carrot getting shredded before subsequently drying in a hot air oven at 50 °C for 24 and 48 h for lettuce and carrot, respectively. Corresponding soil samples from a depth of 0–20 cm were collected using a steel hand auger and subsequently oven-dried (Leblebici & Kar, 2018). The dried samples were homogenized with a ceramic mortar and pestle. The method described by Abbasi and Bahiraei (2012) and Alimohammadi et al. (2020), with slight modification, was used for the digestion of the samples. The acid or wet digestion included a mixture of 10 ml of 69% nitric acid, 5 ml of 65% perchloric acid, and 4 ml of hydrogen peroxide pipetted into an Erlenmeyer flask containing 0.5 g of the finely ground sample, heated for 20 min on a hot stove until a very clear solution was observed. The digested sample was transferred into a 50-ml volumetric flask and made up to 50 ml mark with deionized water. The solution was then filtered with a Whatman No. 1 filter paper, Whatman Ltd, England. The aliquot was subsequently analyzed for the level of the heavy metals of interest with inductively coupled plasma spectrometry (ICP-MS) Nexion 300X, PerkinElmer, USA.

### Quality control

The ICP-MS was calibrated using multielement standard (Agilent Technologies, Japan) with each solution measured three times using external calibration curves for quantification of trace metals. A calibration solution was prepared using ICP-MS multi-element stock standard solution (MERCK) and internal standard to acquire sensitivity factors for individual elements. To determine the accuracy of the results, the NIST CRM 1640 was used as reference material and the recovery rates were subsequently calculated. The limit of detection (LOD) and recovery rate were performed as shown in Table 2. The calibration curves for all elements showed good linearity across the entire concentration range and determination coefficients > 0.989.

**Table 2:** Certified reference material (NIST CRM 1640) for method validation and analytical accuracy

Element	Observed value (mg/kg)	Certified value (mg/kg)	LOD	% of recovery
Cr	0.2 ± 0.01	0.2 ± 0.01	0.04	100
Cu	3.9 ± 0.01	4 ± 0.00	0.4	97.5
Fe	400 ± 32.45	400 ± 0.00	1	100
Ni	1.75 ± 0.02	1.8 ± 0.00	0.1	97
Pb	1.17 ± 0.22	1.2 ± 0.12	0.01	97.5
Zn	52 ± 13.5	55 ± 0.24	1	94.5

### Health risk assessment

The potential health risk associated with heavy metal toxicity from the vegetables is presented in Table 3. The health risk assessment of consuming these vegetables was evaluated by determining the non-carcinogenic health hazard using parameters such as EDI HQ, and HI (Eqs. 1–3), as well as the potential cancer risk exposure (Eq. 4).

**Table 3:** The health risk assessment equation

${\text{EDI}}= \frac{{\text{Cmetal}}\times {\text{IR}}\times {\text{EF}}\times {\text{ED}}}{{\text{BW}}\times {\text{AT}}}$	Eq. 1
${\text{HQ}}= \frac{{\text{EDI}}}{{\text{RfD}}}$	Eq. 2
${\text{HI}}=\sum {\text{HQ}}$	Eq. 3
${\text{LCR}}={\text{EDI}}\times {\text{CSF}}$	Eq. 4

Adapted from Xingmei Liu et al. (2021), Nyambura et al. (2020), Praveena et al. (2018), and Sultana et al. (2017)

For non-carcinogenic health assessment, estimated daily intake (EDI), hazard quotient (HQ), and hazard index (HI) were calculated, while potential cancer risk was calculated by the lifetime cancer risk (LCR) exposure.

EDI is the estimated daily intake of trace metals through vegetables, and RfD is the oral reference dose values for trace metals by Wong et al. (2022). The C*metal* is the determined concentration of metal in vegetable. IR is the daily average ingestion rate of vegetables for adults (0.244 kg/person/day) (Herforth et al., 2019). HQ is the hazard quotient for individual metal in vegetables, while HI is the hazard index, which is the summation of hazard quotients of all the metals. ED is the exposure duration which is given as the average lifespan of an individual (70 years) (Praveena et al., 2018). EF is the exposure frequency given as 365 days/year (Sultana et al., 2017). The BW (60 kg) is the average body weight for adults (Latif et al., 2018). The AT is the average lifespan of an individual (70 years) (Nyambura et al., 2020; Praveena et al., 2018). The CSF is the carcinogenic slope factor from the Integrated Risk Information System (Taiwo et al., 2022).

### Statistical analysis

The data were statistically analyzed using Statistical Package for Social Sciences (IBM-SPSS 28.0). A general linear model multivariate analysis was conducted to determine differences in the mean concentrations of the heavy metals of the two vegetables and the four different types of treatments. The Tukey post hoc homogeneity of variance analysis was used for the separation of means at the alpha level of 0.5.

## Result and discussion

### Heavy metal concentrations in vegetable and soil samples

The effect of organic manures, chemical fertilizers, and types of vegetables on heavy metals accumulation was determined by measuring the concentration of heavy metals in the edible parts of the studied vegetables. The results of the mean concentrations of heavy metals (Cr, Cu, Fe, Ni, Pb, and Zn) in lettuce, carrot, and corresponding soil samples are presented in (Table 4).

**Table 4:** The mean concentrations of heavy metals in vegetables and soil

Treatments	Samples	Trace metals (mg/kg)					
		Cr	Cu	Fe	Ni	Pb	Zn
Untreated soil	Lettuce vegetable	0.02 ± 0.09^a^	1.06 ± 0.31^a^	59.09 ± 15.48^a^	0.24 ± 0.11^a^	0.14 ± 0.01^ab^	7.26 ± 1.55^a^
	Carrot vegetable	0.16 ± 0.04^a^	1.11 ± 0.19^a^	25.09 ± 1.91^a^	0.17 ± 0.02^a^	0.12 ± 0.03^ab^	5.59 ± 0.25^a^
	Lettuce soil	5.80 ± 1.13^b^	2.36 ± 0.35^ab^	1319.31 ± 379^a^	1.42 ± 0.28^bc^	0.80 ± 0.27^cde^	10.06 ± 1.87^ab^
	Carrot soil	5.187 ± 1.26^b^	2.05 ± 0.59^ab^	1251.09 ± 302^a^	1.39 ± 0.38^bc^	0.993 ± 0.81^cde^	16.89 ± 0.23^abc^
Soil + NPK	Lettuce vegetable	0.18 ± 0.04^a^	1.03 ± 0.14^a^	90.89 ± 6.11^a^	0.21 ± 0.03^a^	0.16 ± 0.04^ab^	11.76 ± 0.57^ab^
	Carrot vegetable	0.16 ± 0.09^a^	1.20 ± 0.24^a^	30.37 ± 5.48^a^	0.18 ± 0.03^a^	0.13 ± 0.03^ab^	9.18 ± 1.11^ab^
	Lettuce soil	5.804 ± 1.30^b^	2.03 ± 0.17^ab^	1306.93 ± 131^b^	1.39 ± 0.14^bc^	0.74 ± 0.18^cd^	7.18 ± 0.59^a^
	Carrot soil	5.30 ± 1.10^b^	1.94 ± 0.32^ab^	1141.09 ± 267^b^	1.32 ± 0.16^bc^	0.67 ± 0.13^cd^	6.62 ± 0.58^a^
Soil + cow dung	Lettuce vegetable	0.01h ± 0.03^a^	1.44 ± 0.49^a^	26.96 ± 8.07^a^	0.21 ± 0.04^a^	0.14 ± 0.04^ab^	11.17 ± 1.71^ab^
	Carrot vegetable	0.16 ± 0.05^a^	0.95 ± 0.21^a^	38.77 ± 15.1^a^	0.17 ± 0.02^a^	0.15 ± 0.03^ab^	5.85 ± 0.59^a^
	Lettuce soil	4.86 ± 1.29^b^	1.93 ± 0.32^ab^	1118.71 ± 206^b^	1.22 ± 0.26^b^	0.60 ± 0.12^ab^	6.61 ± 1.04^a^
	Carrot soil	4.43 ± 2.01^b^	1.84 ± 0.52^ab^	994.45 ± 392^b^	1.13 ± 0.41^b^	0.52 ± 0.18^abc^	6.30 ± 1.82^a^
Soil + sewage sludge	Lettuce vegetable	0.20 ± 0.05^a^	3.91 ± 4.33^bc^	73.73 ± 8.07^a^	0.33 ± 0.03^a^	0.12 ± 0.02^a^	20.44 ± 3.78^bc^
	Carrot vegetable	0.13 ± 0.03^a^	2.24 ± 0.29^ab^	32.09 ± 4.38^a^	0.28 ± 0.05^a^	0.10 ± 0.02^a^	13.77 ± 1.64^abc^
	Lettuce soil	5.07 ± 1.03^b^	5.92 ± 0.98^cd^	1167.19 ± 177^b^	1.67 ± 0.26^c^	1.09 ± 0.17^de^	52.42 ± 13.5^d^
	Carrot soil	4.90 ± 0.58^b^	7.76 ± 1.18^d^	1211.74 ± 163^b^	1.73 ± 0.24^c^	1.18 ± 0.10^e^	27.46 ± 4.22^c^

Different exponential letter(s) indicate a statistically significant difference (*p* < 0.05) and the same letter(s) denote no significant difference (*p* > 0.05) of the quoted values in the same column; means were separated via Tukey at alpha level (*p* = 0.05)

There was no significant difference in the mean concentrations of Cr in lettuce and carrot across treatments (*p* > 0.05), with the highest mean concentration of 0.202 ± 0.05 mg/kg plant dry weight recorded for lettuce from sewage sludge. All the values were well below the recommended values in vegetables of 5 mg/kg (Wong et al., 2022). Chromium, in trace amount, is an essential mineral which helps in lipids and carbohydrate metabolism (Abbasi & Bahiraei, 2012). However, it is considered a systemic toxicant that induces multiple organ damage from exposure to high doses, for a long period (Tchounwou et al., 2012). Similarly, there was no significant difference (*p* > 0.05) in the mean concentrations of Fe in both lettuce and carrot across all the treatments in this study. Nevertheless, the highest mean concentration of 90.89 ± 6.11 mg/kg plant dry weight of Fe in vegetable samples was recorded for lettuce from NPK-treated soil. This result supports the suggestion that the high concentration of Fe in vegetables is attributed to its role in chlorophyll synthesis (Li et al., 2021a, 2021b). This may explain why the current study recorded the highest concentration in lettuce, a leafy vegetable and a more chlorophyll-dense vegetable compared to carrot.

The mean concentrations of 3.92 ± 4.33 mg/kg plant dry weight and 2.24 ± 0.29 mg/kg plant dry weight for Cu were recorded for lettuce and carrot from sewage sludge, respectively. These values were significantly higher than the values recorded for the same vegetables from NPK and cow dung treatments. This may suggest a high concentration of the metal in this treatment. Sewage sludge is reported to contain high level of heavy metals due to numerous sources and complex mixtures of its components (Fijalkowski et al., 2017; Raheem et al., 2018).

Even though there was no significant difference (*p* > 0.05) in the mean concentrations of Ni for both lettuce and carrot across different treatments, the highest concentration of 0.33 ± 0.03 mg/kg plant dry weight was recorded for carrot from sewage sludge, and the lowest concentration of 0.17 ± 0.01 mg/kg plant dry weight was observed in lettuce from untreated soil. This result is consistent with the work of Hoaghia et al. (2022) who recorded a higher concentration of Ni in carrot compared to other vegetables. Although the biological functions and nutritional value of Ni in humans are unknown, it has been recognized as an essential nutrient for some microorganisms, plants, and animal species (Song et al., 2017). Long period exposure to high concentration of Ni is known to cause mitochondrial damage due to impairment of mitochondrial membrane potential, reduction of mitochondrial ATP concentration, and destruction of mitochondrial DNA (Genchi et al., 2020).

The highest concentration of Pb with a value of 0.16 ± 0.013 mg/kg plant dry weight was recorded in lettuce from NPK fertilizer treated soil. This value was well below the maximum allowable limit of 0.3 mg/kg for fruit and vegetable by Wong et al. (2022). Comparatively, the result of the present study is lower than those reported by Rehman et al. (2017) and Tewari and Pande (2013). The Agency for Toxic and Disease Registry in 2007 listed Pb as a hazardous element and ranked it second in the world’s top 20 contaminants (ATSDR, 2007). The mean concentrations of Cr, Cu, and Pb in the current study were lower than those reported by Bett et al. (2019) and Tomno et al. (2020) in a similar study from Kenya.

The highest concentration of 20.44 ± 3.78 mg/kg plant dry weight for Zn was observed in lettuce from sewage sludge, which was significantly higher (*p* < 0.05) than the values from other treatments and from carrot. This value is comparable to Ugulu et al. (2021), who also recorded a significantly higher value of 18.385 for Zn compared to other heavy metals and in vegetable grown on press mud treatment. It is believed that the treatment of soil with organic manure enhances the availability of Zn to crops (Wei Wong et al., 2019). The current study recorded the lowest concentration of 5.59 ± 0.25 mg/kg plant dry weight for carrot from untreated soil. The values recorded for Zn by Latif et al. (2018) ranged from 19.5 to 41 mg/kg which were higher than the values recorded in the current study. However, the results of this study were higher than the values reported by Wei Wong et al. (2019) in the review of Zn in vegetables. Nevertheless, the values from this study were all well below the reported maximum allowable limit for Zn in fruits and vegetables (Taiwo et al., 2022). The multiple biological functions of Zn in humans include its roles in nucleic acid metabolism and protein synthesis (Latif et al., 2018).

Comparing the mean concentrations of Cr, Cu, Ni, Pb, and Zn (0.2017, 3.16, 0.3321, 0.1633, and 20.44 mg/kg plant dry weight, respectively) from this study with previous studies showed that our values were significantly higher than those of Wei Wong et al. (2019). However, the values were lower than those of Adedokun et al. (2016) in Nigeria, Azi et al. (2018) from Nigeria, Nabulo et al. (2010) from Uganda, Ngweme et al. (2020) from Congo, and Ali and Al-Qahtani (2012) from Saudi Arabia.

Furthermore, the current study observed the highest concentrations of Cu, Ni, and Zn, heavy metals regarded as nutrients in humans due to their biological functions, in lettuce from sewage sludge treatment. It was observed that lettuce accumulated more metals than carrot, an observation which supports the suggestion that leafy vegetables tend to accumulate more metals than root vegetables (Westbury et al., 2021). It is important to note that there are contradictory findings to this observation. For instance, Hadayat et al. (2018) reported higher accumulation in root vegetables like potato, carrot, and onion compared to leafy vegetables. Conversely, Cherfi et al. (2016) reported that fruit vegetables like tomatoes accumulated more metals than other types of vegetables. This statement directly contradicted Hadayat et al. (2018), who suggested that fruit vegetables or storage organs tend to accumulate less trace metals than other tissue parts of vegetables. Furthermore, the results of Cr, Cu, Ni, and Pb (0.56, 5.20, 2.48, and 0.40 mg/kg, respectively) recorded for carrot, a root vegetable by Hoaghia et al. (2022) in Romania, were higher than those of the current study for both lettuce and carrot.

In this study, application of cow dung and sewage sludge increased the organic matter of the of soil (Table 1) which is understandable as organic matter contents are made from decomposed plant and microbial residues that are heavily present in animal biota and sewage sludge (Osman, 2013). Application of sewage sludge has been reported to increase the soil fertility (Singh & Agrawal, 2007; Liu et al., 2017), and this was evident in the physicochemical properties like organic matter and salinity (EC) of the soil in this study (Table 1).

It has been reported that other than varieties of plants, factors like types and concentrations of contaminants, soil physicochemical properties such as pH, electrical conductivity, and organic matters are also great contributors to the uptake of heavy metals by plants (Aina et al., 2019; Ghani et al., 2022). Soil pH as one of the soil properties determines the quality of agricultural soil and the interaction of other biochemical substances within the soil and the plant. According to Neina (2019), soil pH controls the solubility, mobility, bioavailability, and translocation of heavy metals in plants. It is reported that at low or very acidic pH, heavy metals are usually soluble due to high desorption and very low adsorption, but from intermediate pH (slightly acidic), adsorption increases from no adsorption to almost complete adsorption (Bradl, 2004). As evident in (Table 1) in the current study, it is plausible to suggest that higher concentrations of heavy metals in vegetables from sewage sludge amended soil were also due to the low pH value of the soil.

### Bioaccumulation factor (BAF) of metals of vegetables

**Fig. 1:**
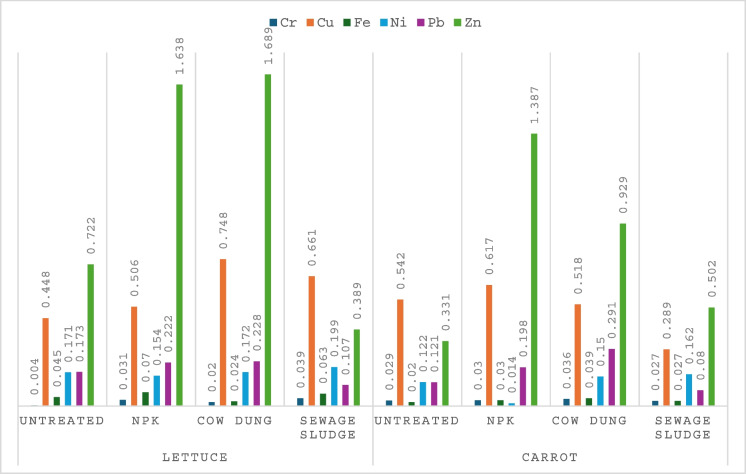
The bioaccumulation factor (BAF) of heavy metals in vegetables

The bioaccumulation factor (BAF) is an estimation of the quantity of heavy metals transferred from the soil into the plant tissue, and it is calculated as the ratio of the concentration of the metal in the plant tissues to its corresponding concentration in the soil. A BAF greater than 1 indicates poor retention of the metals by the soil or high absorption ability of the plant, and vice versa for a BAF less than 1. The BAF is used to categorize plants for their tolerance for heavy metals. It is reported that a BAF < 1 suggests that a plant is a metal excluder, and a BAF > 1 categorizes a plant as a metal accumulator (Olowoyo et al., 2010). Figure 1 presents the results of the BAF for this study. The lowest and the highest BAF values were both recorded for lettuce, with 0.0035 for Cr in lettuce from untreated soil and 1.6889 for Zn in lettuce from cow dung treatments, respectively. The bioaccumulation pattern for lettuce was in the order of Zn > Cu > Pb > Ni > Fe > Cr, while for carrot, it was Zn > Cu > Pb > Ni > Cr > Fe. This result suggests that both lettuce and carrot have a lower tolerance for Cr and Fe.

Also, it was observed the BAF values for Cu, Fe, and Zn were highest in lettuce with only Pb highest in carrot. The result shows that lettuce recorded higher BAF values in 50% of the studied metals (Cu, Fe, Zn), while carrot only recorded higher values in Pb, and there were no differences in the BAF values for Cr and Ni in the two vegetables. The current study is consistent with the study done by Jolly et al. (2013), who reported higher BAF values in leafy vegetables. It has been suggested that leafy vegetables have a high affinity for metal accumulation due to their high transpiration for growth (Sultana et al., 2017). Also, Islam et al. (2016) reported higher BAF for Pb in carrot compared to other types of vegetables in their study. It is believed that types or varieties could influence the BAF of vegetable (Cui et al., 2007); generally, soil factors like pH and salinity are reported to be the most influential factors (Islam et al., 2016). The BAF values were generally low and less than 1 in all the vegetables, apart from Zn in lettuce from NPK and cow dung treatments, as well as carrot from NPK treatment, with values greater than 1. This may suggest that these vegetables are excluders for Cr, Cu, Fe, Ni, and Pb, but have a high tolerance for Zn and could be classified as Zn accumulators.

### Health risk assessment

Table 5 contains the estimated daily intake (EDI) of the six investigated heavy metals for the two investigated vegetables (lettuce and carrot), while the recommended reference dose for the same heavy metals (Taiwo et al., 2022; Wong et al., 2022) is presented in Table 6. The EDI values were generally low, with the lowest value recorded for Cr in lettuce from untreated soil. The pattern of EDI for all the heavy metals was in the order of Fe > Zn > Cu > Pb > Ni > Cr. Soil treatment did not appear to have any effect on the estimated daily intake. However, the result suggests that EDI might be influenced by the type of vegetables, as higher values were recorded for lettuce, a leafy vegetable, compared to carrot, a root vegetable. This observation is consistent with the study by Gupta et al. (2021), where they also recorded higher EDI values in leafy vegetables. The highest daily intake of heavy metals from the consumption of vegetables would be from Fe with a value of 0.37 mg/kg/day. This result is consistent with the findings of Taiwo et al. (2022), who recorded similar EDI values for Fe in fruits and vegetables. Generally, all the EDI values were lower than the recommended maximum daily intake for the metals, which suggests that exposure to metal toxicity from the studied metals in the two vegetables is very unlikely.

**Table 5:** Estimated daily intake of heavy metals from consumption of lettuce and carrot

Vegetables	Treatments	Trace metals (mg/kg/day)					
		Cr	Cu	Fe	Ni	Pb	Zn
Lettuce	Untreated	8.3 × 10^−5^	4.3 × 10^−3^	2.4 × 10^−1^	9.8 × 10^−4^	5.6 × 10^−4^	2.9 × 10^−2^
	NPK	7.3 × 10^−4^	4.2 × 10^−3^	3.7 × 10^−1^	8.7 × 10^−4^	6.6 × 10^−4^	4.8 × 10^−2^
	Cow dung	3.9 × 10^−4^	5.8 × 10^−3^	1.1 × 10^−1^	8.6 × 10^−4^	5.5 × 10^−4^	4.5 × 10^−2^
	Sewage sludge	8.2 × 10^−4^	1.6 × 10^−3^	3.0 × 10^−1^	1.4 × 10^−4^	4.7 × 10^−4^	8.3 × 10^−2^
Carrot	Untreated	6.3 × 10^−4^	4.5 × 10^−3^	1.0 × 10^−1^	6.9 × 10^−4^	4.9 × 10^−4^	2.2 × 10^−2^
	NPK	6.5 × 10^−4^	4.8 × 10^−3^	1.2 × 10^−1^	7.4 × 10^−4^	5.4 × 10^−4^	3.7 × 10^−2^
	Cow dung	6.5 × 10^−4^	3.8 × 10^−3^	1.6 × 10^−1^	6.9 × 10^−4^	6.1 × 10^−4^	2.4 × 10^−2^
	Sewage sludge	5.4 × 10^−4^	9.1 × 10^−3^	1.3 × 10^−1^	1.1 × 10^−4^	3.8 × 10^−4^	5.6 × 10^−2^

**Table 6:** The reference dose for heavy metals (RfD)

Trace metals	RfD mg/kg
Cr	0.003
Cu	0.04
Fe	0.7
Ni	0.02
Pb	0.0035
Zn	0.3

Adapted from Wong et al. (2022) and Taiwo et al. (2022)

### The non-carcinogenic health effects

**Fig. 2:**
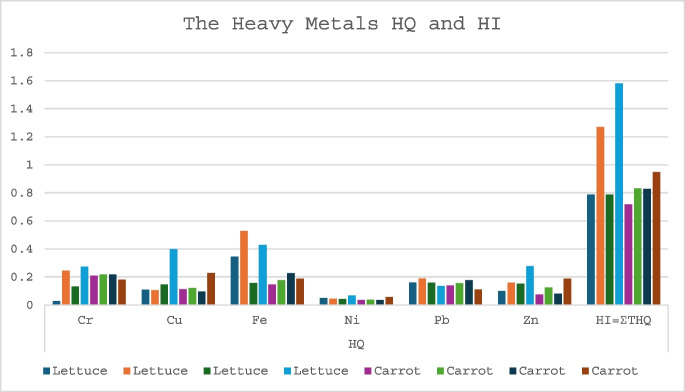
The hazard quotient and hazard index of heavy metals in lettuce and carrot

The result of non-carcinogenic health risk calculated from the hazard quotient (HQ) for individual metals and the total hazard quotient for all the metals, known as hazard index (HI), is presented in Fig. 2. The HQ values varied from 0.028 for Cr in lettuce from untreated soil to 0.528 for Fe in lettuce from NPK treatment. The HQ for each metal was less than the allowable maximum threshold of 1, suggesting intake of a single metal from the consumption of vegetables does not pose a health threat (Hawrami et al., 2020; Tariq, 2021; Xu et al., 2016). However, the HI, which is the sum of the HQ values for all metals in the vegetables, suggested exposure to a potential health hazard.

The HI values of 1. 269 and 1.580 recorded for lettuce from NPK and municipal sewage sludge treatments, respectively, indicate that consuming lettuce from these two treatments may be harmful and result in potential non-carcinogenic diseases.

### The potential carcinogenic health risk

The probability of an individual developing cancer due to daily exposure to carcinogenic metals over a lifetime period, from the consumption of the studied vegetables, was evaluated and presented in Table 7. This was evaluated using the guidelines for carcinogenic risk assessment by the Environmental Protection Agency (EPA, 2005). Only the carcinogenic risk associated with Cr and Pb with CSF 0.5 and 0.0085, respectively, was evaluated. The other classified carcinogenic heavy metals like As and Cd from our investigated metals were below detection in this study. The results of the current study range from 4.2 × 10^−8^ to 5.6 × 10^−6^. According to USEPA, (2011), the carcinogenic risk factor that is < 10^−6^ is negligible and considered not a concern, and a factor between 10^−6^ and 10^−4^ is permissible, but a factor > 10^−4^ is considered unacceptable and indicates a potential cancer risk. The values in the current study range from 10^−8^ for Cr in lettuce from untreated soil to 10^−6^ for Pb in lettuce from NPK treated soil. These values are in the negligible range of cancer risk exposure from heavy metal ingestion. It suggests that using these methods of soil treatments in lettuce and carrot production may not expose consumers to cancer development.

**Table 7:** Estimated potential lifetime cancer risk

Vegetables	Treatments	Cr	Pb
Lettuce	Untreated soil	4.2 × 10^−8^	4.8 × 10^−6^
	NPK	3.7 × 10^−7^	5.6 × 10^−6^
	Cow dung	2.0 × 10^−7^	4.7 × 10^−6^
	Sewage sludge	4.1 × 10^−7^	4.0 × 10^−6^
Carrot	Untreated soil	3.2 × 10^−7^	4.2 × 10^−6^
	NPK	3.3 × 10^−7^	4.6 × 10^−6^
	Cow dung	3.3 × 10^−7^	5.2 × 10^−6^
	Sewage Sludge	2.7 × 10^−7^	3.2 × 10^−6^

## Conclusion

In conclusion, the present study revealed that although lettuce and carrot cultivated on sewage sludge cow dung and NPK fertilized soil might not necessarily expose consumers to potential development cancer for now, there are however other metal health hazards that should be of a great concern. However, the HI results indicated that consuming lettuce from NPK and sewage sludge may expose consumers to a toxic metal-related non-carcinogenic health problem. Therefore, the choice of soil treatments should be dependent on the types of vegetables to be cultivated. It is imperative that the production vegetables receive strict monitoring by the appropriate policymakers. Even though numerous research studies have investigated types of soil management agricultural production, most of these studies have majorly centered on the effects of soil treatments on the nutritional quality of food crops. Since the current study only looked at a few heavy metals and two types of vegetables, this study therefore recommends continuous research into potential health risks from different soil treatments used in vegetable production. This study has opened further research into the microbial quality assessment of vegetables cultivated with these soil amendment types.

## Data Availability

All data generated or analyzed during this study are included in this published article. Should any raw data files be needed in another format, they are available from the corresponding author upon reasonable request.

## References

[CR1] Abbasi S, Bahiraei A (2012). Ultra trace quantification of chromium(VI) in food and water samples by highly sensitive catalytic adsorptive stripping voltammetry with rubeanic acid. Food Chemistry.

[CR2] Adani P, Sawale AA, Nandhagopal G (2022). Bioaccumulation of heavy metals in the food components from water and sediments in the coastal waters of Kalpakkam, Southeast coast of India. Environmental Nanotechnology, Monitoring & Management.

[CR3] Adedokun AH, Njoku KL, Akinola MO, Adesuyi AA, Jolaoso AO (2016). Potential human health risk assessment of heavy metals intake via consumption of some leafy vegetables obtained from four market in Lagos Metropolis, Nigeria. Journal of Applied Sciences and Environmental Management.

[CR4] Aina OE, Amoo SO, Mugivhisa LL, Olowoyo JO (2019). Effect of organic and inorganic sources of nutrients on the bioactive compounds and antioxidant activity of tomato. Applied Ecology and Environmental Research.

[CR5] Aina, O. E., Olowoyo, J. O., Mugivhisa, L. L. & Amoo, S. O. (2018). *Effect of different soil amendments on growth performance and levels of copper and zinc in Lycopersicon esculentum*. www.neptjournal.com. Accessed 12 Nov 2023

[CR6] Ali MHH, Al-Qahtani KM (2012). Assessment of some heavy metals in vegetables, cereals and fruits in Saudi Arabian markets. The Egyptian Journal of Aquatic Research.

[CR7] Alimohammadi M, Younesian M, Madihi-Bidgoli S, Nabizadeh Nodehi R, Jahed Khaniki GR, Hadi M, Ghanbari F (2020). Heavy metal(oid)s concentration in Tehran supermarket vegetables: Carcinogenic and non-carcinogenic health risk assessment*. Toxin Reviews.

[CR8] Ansorena MR, Agüero MV, Goñi MG, Roura S, Ponce A, del Rosario Moreira M, Di Scala K (2012). Assessment of lettuce quality during storage at low relative humidity using Global Stability Index methodology. Food Science and Technology.

[CR9] ATSDR. (2007). *Priority list of hazardous substances: Agency for Toxic Substances and Disease Registry*. https://www.atsdr.cdc.gov/spl/previous/07list.html. Accessed 11 Dec 2023

[CR10] Aylaj M, Lhadi EK, Adani F (2019). Municipal waste and poultry manure compost affect biomass production, nitrate reductase activity and heavy metals in tomato plants. Compost Science & Utilization.

[CR11] Azi F, Odo MO, Okorie PA, Njoku HA, Nwobasi VN, David E, Onu TC (2018). Heavy metal and microbial safety assessment of raw and cooked pumpkin and *Amaranthus*
*viridis* leaves grown in Abakaliki, Nigeria. Food Science & Nutrition.

[CR12] Beraki, A. F., Landman, W. A., DeWitt, D. G., Olivier, C., Mathole, K., Ndarana, T., & South Africa Water Research Commission. (2013). *Modelled sea-surface temperature scenario considerations and Southern African seasonal rainfall and temperature predictability: Report to the Water Research Commission*. https://www.academia.edu/download/49957772/Modelled_Sea-Surface_Temperature_Scenari20161029-6066-mhxwb7.pdf

[CR13] Bett, L., Gilbert, O., Phanice, W., & Mule, S. (2019). Determination of some heavy metals in soils and vegetables samples from Kericho West Sub-county, Kenya. *Chemical Science International Journal*, 1–10. 10.9734/csji/2019/v28i230134

[CR14] Bradl HB (2004). Adsorption of heavy metal ions on soils and soils constituents. Journal of Colloid and Interface Science.

[CR15] Chauhan P, Mathur J (2020). Phytoremediation efficiency of Helianthus annuus L. for reclamation of heavy metals-contaminated industrial soil. Environmental Science and Pollution Research.

[CR16] Cherfi A, Cherfi M, Maache-Rezzoug Z, Rezzoug S-A (2016). Risk assessment of heavy metals via consumption of vegetables collected from different supermarkets in La Rochelle, France. Environmental Monitoring and Assessment.

[CR17] Cruz R, Gomes T, Ferreira A, Mendes E, Baptista P, Cunha S, Pereira JA, Ramalhosa E, Casal S (2014). Antioxidant activity and bioactive compounds of lettuce improved by espresso coffee residues. Food Chemistry.

[CR18] Cui K, Shoemaker SP (2018). A look at food security in China. Npj Science of Food.

[CR19] Cui S, Zhou Q, Chao L (2007). Potential hyperaccumulation of Pb, Zn, Cu and Cd in endurant plants distributed in an old smeltery, northeast China. Environmental Geology.

[CR20] Daliakopoulos IN, Tsanis IK, Koutroulis A, Kourgialas NN, Varouchakis AE, Karatzas GP, Ritsema CJ (2016). The threat of soil salinity: A European scale review. Science of the Total Environment.

[CR21] DALRRD. (2021a). *Department of Agriculture, Land Reform and Rural Development: A profile of the South African carrot market value chain 2021*. www.daff.gov.za. Accessed 20 Mar 2024

[CR22] DALRRD. (2021b). *Department of Agriculture, Land Reform and Rural Development: A profile of the South African lettuce market value chain 2021*. www.dalrrd.gov.za. Accessed 20 Mar 2020

[CR23] EPA. (2005). *Guidelines for carcinogen risk assessment: Environmental Protection Agency*. https://www3.epa.gov/airtoxics/cancer_guidelines_final_3-25-05.pdf. Accessed 15 Mar 2024

[CR24] Esposito M, De Roma A, Cavallo S, Miedico O, Chiaravalle E, Soprano V, Baldi L, Gallo P (2019). Trace elements in vegetables and fruits cultivated in Southern Italy. Journal of Food Composition and Analysis.

[CR25] FAO., IFAD., UNICEF., WFP., & WHO. (2021). *The state of food security and nutrition in the world 2021. Transforming food systems for food security, improved nutrition and affordable healthy diets for all. Rome, FAO.* FAO, IFAD, UNICEF, WFP and WHO. 10.4060/cb4474en

[CR26] Fijalkowski K, Rorat A, Grobelak A, Kacprzak MJ (2017). The presence of contaminations in sewage sludge – The current situation. Journal of Environmental Management.

[CR27] Genchi G, Carocci A, Lauria G, Sinicropi MS, Catalano A (2020). Nickel: Human health and environmental toxicology. International Journal of Environmental Research and Public Health.

[CR28] Ghani J, Nawab J, Khan S, Khan MA, Ahmad I, Ali HM, Siddiqui MH, Funari V, Dinelli E (2022). Organic amendments minimize the migration of potentially toxic elements in soil–plant system in degraded agricultural lands. Biomass Conversion and Biorefinery.

[CR29] Głąbska D, Guzek D, Groele B, Gutkowska K (2020). Fruit and vegetable intake and mental health in adults: A systematic review. Nutrients.

[CR30] Goss, M. J., Tubeileh, A., & Goorahoo, D. (2013). *A review of the use of organic amendments and the risk to human health* (pp. 275–379). 10.1016/B978-0-12-407686-0.00005-1

[CR31] Guadie A, Yesigat A, Gatew S, Worku A, Liu W, Ajibade FO, Wang A (2021). Evaluating the health risks of heavy metals from vegetables grown on soil irrigated with untreated and treated wastewater in Arba Minch, Ethiopia. Science of the Total Environment.

[CR32] Gupta S, Jyothi Lakshmi A, Manjunath MN, Prakash J (2005). Analysis of nutrient and antinutrient content of underutilized green leafy vegetables. LWT - Food Science and Technology.

[CR33] Gupta N, Yadav KK, Kumar V, Krishnan S, Kumar S, Nejad ZD, Majeed Khan MA, Alam J (2021). Evaluating heavy metals contamination in soil and vegetables in the region of North India: Levels, transfer and potential human health risk analysis. Environmental Toxicology and Pharmacology.

[CR34] Hadayat N, De Oliveira LM, Da Silva E, Han L, Hussain M, Liu X, Ma LQ (2018). Assessment of trace metals in five most-consumed vegetables in the US: Conventional vs. organic. Environmental Pollution.

[CR35] Hamdi H, Hechmi S, Khelil MN, Zoghlami IR, Benzarti S, Mokni-Tlili S, Hassen A, Jedidi N (2019). Repetitive land application of urban sewage sludge: Effect of amendment rates and soil texture on fertility and degradation parameters. CATENA.

[CR36] Hammad HM, Khaliq A, Abbas F, Farhad W, Fahad S, Aslam M, Shah GM, Nasim W, Mubeen M, Bakhat HF (2020). Comparative effects of organic and inorganic fertilizers on soil organic carbon and wheat productivity under arid region. Communications in Soil Science and Plant Analysis.

[CR37] Hawrami KAM, Crout NMJ, Shaw G, Bailey EH (2020). Assessment of potentially toxic elements in vegetables cultivated in urban and peri-urban sites in the Kurdistan region of Iraq and implications for human health. Environmental Geochemistry and Health.

[CR38] He J, Wu H, Hu W, Liu J, Zhang Q, Xiao W, Hu M, Wu M, Huang F (2022). Exposure to multiple trace elements and thyroid cancer risk in Chinese adults: A case-control study. International Journal of Hygiene and Environmental Health.

[CR39] Herforth A, Arimond M, Álvarez-Sánchez C, Coates J, Christianson K, Muehlhoff E (2019). A global review of food-based dietary guidelines. Advances in Nutrition.

[CR40] Hoaghia M-A, Cadar O, Moisa C, Roman C, Kovacs E (2022). Heavy metals and health risk assessment in vegetables grown in the vicinity of a former non-metallic facility located in Romania. Environmental Science and Pollution Research.

[CR41] Islam MdS, Ahmed MdK, Habibullah-Al-Mamun Md (2016). Apportionment of heavy metals in soil and vegetables and associated health risks assessment. Stochastic Environmental Research and Risk Assessment.

[CR42] Jiao W, Chen W, Chang AC, Page AL (2012). Environmental risks of trace elements associated with long-term phosphate fertilizers applications: A review. Environmental Pollution.

[CR43] Jolly YN, Islam A, Akbar S (2013). Transfer of metals from soil to vegetables and possible health risk assessment. SpringerPlus.

[CR44] Khan S, Reid BJ, Li G, Zhu Y-G (2014). Application of biochar to soil reduces cancer risk via rice consumption: A case study in Miaoqian village, Longyan, China. Environment International.

[CR45] Khan, M. N., Mobin, M., Abbas, Z. K., & Alamri, S. A. (2018). Fertilizers and their contaminants in soils, surface and groundwater. In *Encyclopedia of the Anthropocene* (pp. 225–240). Elsevier. 10.1016/B978-0-12-809665-9.09888-8

[CR46] Kumar Bhatt M, Labanya R, Joshi HC (2019). Influence of long-term chemical fertilizers and organic manures on soil fertility - A review. Universal Journal of Agricultural Research.

[CR47] Latif, A., Bilal, M., Asghar, W., Azeem, M., Ahmad, M. I., Abbas, A., Zulfiqar Ahmad, M., & Shahzad, T. (2018). Heavy metal accumulation in vegetables and assessment of their potential health risk. *Journal of Environmental Analytical Chemistry*, *05*(01). 10.4172/2380-2391.1000234

[CR48] Leblebici, Z., & Kar, M. (2018). Heavy metals accumulation in vegetables irrigated with different water sources and their human daily intake in Nevsehir. *Journal of Agricultural Science and Technology, 20*. http://hdl.handle.net/20.500.11787/4812

[CR49] Li S, Sun X, Li S, Liu Y, Ma Q, Zhou W (2021). Effects of amendments on the bioavailability, transformation and accumulation of heavy metals by pakchoi cabbage in a multi-element contaminated soil. RSC Advances.

[CR50] Li, J., Cao, X., Jia, X., Liu, L., Cao, H., Qin, W., & Li, M. (2021a). Iron deficiency leads to chlorosis through impacting chlorophyll synthesis and nitrogen metabolism in Areca catechu L. *Frontiers in Plant Science*, *12*. 10.3389/fpls.2021.710093 10.3389/fpls.2021.710093 PMC8365612 34408765

[CR51] Liu R, Pieniak Z, Verbeke W (2013). Consumers’ attitudes and behaviour towards safe food in China: A review. Food Control.

[CR52] Liu X, Liu W, Wang Q, Wu L, Luo Y, Christie P (2017). Soil properties and microbial ecology of a paddy field after repeated applications of domestic and industrial sewage sludges. Environmental Science and Pollution Research.

[CR53] Liu X, Gu S, Yang S, Deng J, Xu J (2021). Heavy metals in soil-vegetable system around E-waste site and the health risk assessment. Science of the Total Environment.

[CR54] Lyon, F. (2010). *IARC monographs on the evaluation of carcinogenic risks to humans VOLUME 94 Ingested Nitrate and Nitrite, and Cyanobacterial Peptide Toxins*. https://monographs.iarc.who.int/wp-content/uploads/2018/08/14-002.pdf PMC4781178 21141240

[CR55] Menni C, Louca P, Berry SE, Vijay A, Astbury S, Leeming ER, Gibson R, Asnicar F, Piccinno G, Wolf J, Davies R, Mangino M, Segata N, Spector TD, Valdes AM (2021). High intake of vegetables is linked to lower white blood cell profile and the effect is mediated by the gut microbiome. BMC Medicine.

[CR56] Nabulo G, Young SD, Black CR (2010). Assessing risk to human health from tropical leafy vegetables grown on contaminated urban soils. Science of the Total Environment.

[CR57] Nawab J, Khan N, Ahmed R, Khan S, Ghani J, Rahman Z, Khan F, Wang X, Muhammad J, Sher H (2019). Influence of different organic geo-sorbents on Spinacia oleracea grown in chromite mine-degraded soil: A greenhouse study. Journal of Soils and Sediments.

[CR58] Neina D (2019). The role of soil pH in plant nutrition and soil remediation. Applied and Environmental Soil Science.

[CR59] Ngweme GN, Atibu EK, Al Salah DMM, Muanamoki PM, Kiyombo GM, Mulaji CK, Otamonga J-P, Poté JW (2020). Heavy metal concentration in irrigation water, soil and dietary risk assessment of Amaranthus viridis grown in peri-urban areas in Kinshasa, Democratic Republic of the Congo. Watershed Ecology and the Environment.

[CR60] Ning C, Gao P, Wang B, Lin W, Jiang N, Cai K (2017). Impacts of chemical fertilizer reduction and organic amendments supplementation on soil nutrient, enzyme activity and heavy metal content. Journal of Integrative Agriculture.

[CR61] Nyambura C, Hashim NO, Chege MW, Tokonami S, Omonya FW (2020). Cancer and non-cancer health risks from carcinogenic heavy metal exposures in underground water from Kilimambogo, Kenya. Groundwater for Sustainable Development.

[CR62] Olowoyo JO, van Heerden E, Fischer JL, Baker C (2010). Trace metals in soil and leaves of Jacaranda mimosifolia in Tshwane area, South Africa. Atmospheric Environment.

[CR63] Olowoyo JO, Okedeyi OO, Mkolo NM, Lion GN, Mdakane STR (2012). Uptake and translocation of heavy metals by medicinal plants growing around a waste dump site in Pretoria, South Africa. South African Journal of Botany.

[CR64] Osman, K. T. (2013). Soil organic matter. In *Soils* (pp. 89–96). Springer Netherlands. 10.1007/978-94-007-5663-2_7

[CR65] Pipoyan D, Stepanyan S, Beglaryan M, Stepanyan S, Asmaryan S, Hovsepyan A, Merendino N (2020). Carcinogenic and non-carcinogenic risk assessment of trace elements and POPs in honey from Shirak and Syunik regions of Armenia. Chemosphere.

[CR66] Praveena SM, Pradhan B, Aris AZ (2018). Assessment of bioavailability and human health exposure risk to heavy metals in surface soils (Klang district, Malaysia). Toxin Reviews.

[CR67] Raheem A, Sikarwar VS, He J, Dastyar W, Dionysiou DD, Wang W, Zhao M (2018). Opportunities and challenges in sustainable treatment and resource reuse of sewage sludge: A review. Chemical Engineering Journal.

[CR68] Rehman ZU, Khan S, Brusseau ML, Shah MT (2017). Lead and cadmium contamination and exposure risk assessment via consumption of vegetables grown in agricultural soils of five-selected regions of Pakistan. Chemosphere.

[CR69] Shah F, Wu W (2019). Soil and crop management strategies to ensure higher crop productivity within sustainable environments. Sustainability.

[CR70] Siedt M, Schäffer A, Smith KEC, Nabel M, Roß-Nickoll M, van Dongen JT (2021). Comparing straw, compost, and biochar regarding their suitability as agricultural soil amendments to affect soil structure, nutrient leaching, microbial communities, and the fate of pesticides. Science of the Total Environment.

[CR71] Singh RP, Agrawal M (2007). Effects of sewage sludge amendment on heavy metal accumulation and consequent responses of Beta vulgaris plants. Chemosphere.

[CR72] Singh, T. B., Ali, A., Prasad, M., Yadav, A., Shrivastav, P., Goyal, D., & Dantu, P. K. (2020). Role of organic fertilizers in improving soil fertility. In *Contaminants in Agriculture* (pp. 61–77). Springer International Publishing. 10.1007/978-3-030-41552-5_3

[CR73] Song X, Fiati Kenston SS, Kong L, Zhao J (2017). Molecular mechanisms of nickel induced neurotoxicity and chemoprevention. Toxicology.

[CR74] Sultana MS, Rana S, Yamazaki S, Aono T, Yoshida S (2017). Health risk assessment for carcinogenic and non-carcinogenic heavy metal exposures from vegetables and fruits of Bangladesh. Cogent Environmental Science.

[CR75] Taiwo AM, Olowookere ZA, Bada BS, Akinhanmi TF, Oyedepo JA (2022). Contamination and health risk assessments of metals in selected fruits from Abeokuta, Southwestern Nigeria. Journal of Food Composition and Analysis.

[CR76] Tariq FS (2021). Heavy metals concentration in vegetables irrigated with municipal wastewater and their human daily intake in Erbil city. Environmental Nanotechnology, Monitoring & Management.

[CR77] Tchounwou, P. B., Yedjou, C. G., Patlolla, A. K., & Sutton, D. J. (2012). *Heavy metal toxicity and the environment* (pp. 133–164). 10.1007/978-3-7643-8340-4_6 10.1007/978-3-7643-8340-4_6 PMC4144270 22945569

[CR78] Tewari, G. & Pande, C. (2013). African journal of agricultural research health risk assessment of heavy metals in seasonal vegetables from north-west Himalaya*. 8*(23), 3019–3024.10.5897/AJAR2013.6955

[CR79] Thompson RB, Incrocci L, van Ruijven J, Massa D (2020). Reducing contamination of water bodies from European vegetable production systems. Agricultural Water Management.

[CR80] Tomno RM, Nzeve JK, Mailu SN, Shitanda D, Waswa F (2020). Heavy metal contamination of water, soil and vegetables in urban streams in Machakos municipality, Kenya. Scientific African.

[CR81] Ugulu I, Ahmad K, Khan ZI, Munir M, Wajid K, Bashir H (2021). Effects of organic and chemical fertilizers on the growth, heavy metal/metalloid accumulation, and human health risk of wheat (Triticum aestivum L.). Environmental Science and Pollution Research.

[CR82] Unver, M. C., Ugulu, I., Durkan, N., Baslar, S., & Dogan, Y. (2015). Heavy metal contents of Malva sylvestris sold as edible greens in the local markets of Izmir. *Ekoloji*, 13–25. 10.5053/ekoloji.2015.01

[CR83] USEPA. (2011). *Framework for metals risk assessment: United States Environmental Protection Agency*. www.epa.gov/osa. Accessed 15 Mar 2024

[CR84] Uusiku NP, Oelofse A, Duodu KG, Bester MJ, Faber M (2010). Nutritional value of leafy vegetables of sub-Saharan Africa and their potential contribution to human health: A review. Journal of Food Composition and Analysis.

[CR85] Vácha R (2021). Heavy metal pollution and its effects on agriculture. Agronomy.

[CR86] Wei Wong, K., Kong Yap, C., Nulit, R., Omar, H., Zaharin Aris, A., Hee Cheng, W., Talib Latif, M., & Seng Leow, C. (2019). Zn in vegetables: A review and some insights. *Integrative Food, Nutrition and Metabolism*, *6*(2). 10.15761/IFNM.1000245

[CR87] Westbury S, Ghosh I, Jones HM, Mensah D, Samuel F, Irache A, Azhar N, Al-Khudairy L, Iqbal R, Oyebode O (2021). The influence of the urban food environment on diet, nutrition and health outcomes in low-income and middle-income countries: A systematic review. BMJ Global Health.

[CR88] Wong C, Roberts SM, Saab IN (2022). Review of regulatory reference values and background levels for heavy metals in the human diet. Regulatory Toxicology and Pharmacology.

[CR89] Xu F, Qiu L, Cao Y, Huang J, Liu Z, Tian X, Li A, Yin X (2016). Trace metals in the surface sediments of the intertidal Jiaozhou Bay, China: Sources and contamination assessment. Marine Pollution Bulletin.

[CR90] Zhou H, Yang W-T, Zhou X, Liu L, Gu J-F, Wang W-L, Zou J-L, Tian T, Peng P-Q, Liao B-H (2016). Accumulation of heavy metals in vegetable species planted in contaminated soils and the health risk assessment. International Journal of Environmental Research and Public Health.

